# Association of objectively measured sedentary behavior and physical activity with cardiometabolic risk markers in older adults

**DOI:** 10.1371/journal.pone.0210861

**Published:** 2019-01-18

**Authors:** Thamara Hübler Figueiró, Gabriel Claudino Budal Arins, Carla Elane Silva dos Santos, Francieli Cembranel, Paulo Adão de Medeiros, Eleonora d’Orsi, Cassiano Ricardo Rech

**Affiliations:** 1 Postgraduate Program in Collective Health, Federal University of Santa Catarina, Florianópolis, Santa Catarina, Brazil; 2 Postgraduate Program in Physical Education, Federal University of Santa Catarina, Florianópolis, Santa Catarina, Brazil; 3 Department of Public Health, Federal University of Santa Catarina, Florianópolis, Santa Catarina, Brazil; 4 The Bernard Lown Scholars in Cardiovascular Health Program, Department of Global Health and Population, Harvard T.H. Chan School of Public Health, Boston, USA; 5 Department of Physical Education, Federal University of Santa Catarina, Florianópolis, Santa Catarina, Brazil; Universidad Pablo de Olavide, SPAIN

## Abstract

**Objective:**

The aim of this study was to examine the associations between sedentary behavior and different intensities of physical activity with cardiometabolic risk, and to analyze the simultaneous effect of excess sedentary behavior and recommended levels of physical activity on cardiometabolic risk markers in older adults.

**Methods:**

We conducted a population-based cross-sectional study on a sample of older adults (60+) living in Florianopolis, Brazil. The objectively measured predictors were sedentary time, light physical activity and moderate to vigorous physical activity, and the outcomes were markers of cardiometabolic risk. Data were considered valid when the participant had used the accelerometer for at least four days per week.

**Results:**

The sample included 425 older adults (59.8% women), with a mean age of 73.9 years (95%CI: 73.5–74.4). Sedentary behavior was associated with lower systolic blood pressure levels (β = -0.03; 95%CI: -0.05; -0.01) and lower HDL cholesterol (β = -0.02; 95%CI: -0.02; -0.01). Light physical activity was not associated with any cardiovascular risk markers after adjustment. Each minute spent in moderate to vigorous physical activity was associated with lower waist circumference (β = -0.15; 95%CI: -0.24; -0.05), systolic blood pressure (β = -0.18; 95%CI: -0.32; -0.04) and plasma glucose (β = -0.18; 95%CI: -0.33;-0.02), and with higher HDL cholesterol (β = 0.10; 95%CI: 0.01; 0.18). Moreover, physically inactive and sedentary individuals had a greater mean waist circumference and lower HDL cholesterol than physically active and non-sedentary subjects.

**Conclusion:**

The results suggest that moderate to vigorous physical activity have a positive impact on cardiometabolic risk markers in older adults. Light physical activity does not appear to have a beneficial effect on the cardiometabolic markers, and despite the benefits provided by the different intensities of physical activity, the simultaneous presence of sedentary behavior and low physical activity level was associated with poor cardiometabolic risk markers.

## Introduction

Cardiovascular diseases (CVD) are a leading cause of death in the world and represent an important public health problem [1]. In 2015, about 28% of deaths in the Americas were attributed to CVD. Among the elderly, two out of five deaths are caused by this group of diseases [2]. The risk of CVD is higher among individuals with altered cardiometabolic indicators, such as a greater waist circumference (WC) [3], higher blood pressure [4] and high concentrations of low-density lipoprotein cholesterol (LDL-c) [5].

Evidence indicates that these changes in cardiometabolic risk markers could be minimized with regular physical activity (PA) [6–8] and reduced sedentary behavior (SB) [9]. Recent studies have shown that adults who remain active have lower values of WC, LDL-c and triglycerides [10,11]. However, the results obtained for the elderly population have been inconsistent [12,13].

Despite the importance of the practice of PA as a protection factor against CVD [14], the associations seem to be dependent on the intensity of PA [15,16]. Performing moderate to vigorous physical activity (MVPA) provides greater protection against CVD when compared to light physical activity (LPA) [17]. However, older adults spend less time in MVPA, with approximately 80% of them being insufficiently active, i.e., they do not reach the recommendations to ensure health benefits (≥ 150 min/week of MVPA) [18]. On the other hand, older adults tend to spend more time in LPA, but few studies have addressed activities performed at this intensity as a factor protecting against CVD [18,19]. In adults, LPA has been shown to be independently associated with lower triglyceride levels, lipid accumulation, and insulin resistance [20]. It is therefore important to analyze the association between LPA and cardiometabolic risk markers in the elderly population.

Similarly, inconsistent results were observed regarding the effect of SB on cardiometabolic risk markers in older adults [9]. Sedentary behavior is defined as any waking behavior characterized by an energy expenditure of 1.5 METs or less in the sitting, reclining or lying position [21]. Evidence shows that most older adults spend more than eight hours per day in SB [22] but the results of studies investigating the association between this behavior and triglycerides, high-density lipoprotein cholesterol (HDL-c), blood pressure, blood glucose and total cholesterol are inconclusive [9].

In addition to the individual effects of different levels of PA and SB, older adults generally exhibited both reduced levels of PA and a large amount of time spent in SB [18–22]. However, the simultaneous effect of complying with PA recommendations and time spent in SB is still poorly explored in the elderly population. It is believed that part of the inconsistencies in the evidence regarding the elderly is due to the small number of studies and to the lack of data of objectively measured PA and SB that would allow to more accurately estimate their intensities since subjective evaluation of these measures is more common, producing less reliable results. Thus, this study advances the use of objective measures of PA and SB evaluated by accelerometry to analyze the relationships between PA and cardiometabolic risk factors in the older adult population.

The specific aims of this study were 1) to examine the association of SB and different intensities of PA measured by accelerometry with cardiometabolic risk markers, and 2) to analyze the simultaneous effect of excess SB (> 10 h/day) and complying with PA recommendations (≥ 150 min/week) on cardiometabolic risk markers in older adults.

## Methods

This was a cross-sectional study that used data from the second wave of the “Health Conditions of Older Adults in Florianópolis (EpiFloripa Idoso Study)” conducted in Florianópolis, Santa Catarina, Southern Brazil. The study included older adults aged 60 years and older of both genders living in the urban area. The city is the capital of Santa Catarina state and is characterized by a high Human Development Index compared to the national Brazilian average (0.847 versus 0.755) [23].

The baseline data of the EpiFloripa Idoso Study were collected in 2009/2010 (n = 1,702) and the follow-up data in 2013/2014 (n = 1,197). The baseline (2009/10) sampling method used was two-stage clustering. In the first stage, 80 of 420 census tracts were systematically selected considering the average monthly household income. In the second stage, the households were the units. Sectors with fewer than 150 households were grouped and those with more than 500 households (according to the corresponding income decile) were divided, resulting in 83 census sectors. The sample size was calculated based on the prevalence that generated the maximum data variability (50%), a margin of error of 4 percentage points, and a confidence level of 95%. The sample was doubled to correct for the design effect. An extra 35% was added because of the expected non-response rate and to control for confounding variables in the multivariate analysis. Therefore, the final sample included 1,599 older adults. Details of the sample are described elsewhere [24].

For follow-up, an active search aimed at recruiting all participants in the baseline study was done using telephone contacts, letters, posters, electronic media, and health information systems. Among 1,702 respondents in the first wave, there were 376 losses (22.1%) that included 217 deaths and 129 refusals (7.6%), totaling 1,197 participants in 2013/2014 (response rate of 70.3%) [24].

### Sub-sample

In 2013/14, the participants were invited by telephone to undergo clinical and imaging examinations and monitoring tests. Subjects who refused to undergo the tests after three unsuccessful telephone contacts were considered refusals and those who did not attend the examinations after the third scheduled visit were considered losses. The sub-sample consisted of 604 participants. All procedures were approved by the Ethics Committee on Research Involving Humans of the Federal University of Santa Catarina [protocol number 352/2008 on 15/12/2008; follow-up (protocol number 329.650 on 08/07/2013); clinical examinations (protocol number 526.126 on 09/12/2013, CAAE No. 16731313.0.0000.0121)]. Written informed consent was obtained from all participants prior to the interview and clinical examinations.

### Cardiometabolic risk markers

The outcomes were cardiometabolic risk markers and included WC, systolic blood pressure (SBP), diastolic blood pressure (DBP), fasting plasma glucose, HDL-c, LDL-c, and triglycerides.

Blood pressure and WC were obtained in the second interview (2013/2014) at the participant’s home. The WC was measured in the narrowest part of the abdomen below the last rib. When the narrowest part could not be found, the midpoint between the lower costal margin and iliac crest was evaluated during expiration. The measurement was performed twice with a 200-cm inelastic measuring tape (variation of 0.1 cm). When the error between the two measurements was ≥ 1 cm, a third measurement was taken and the average of the two closest measurements was used for analysis. Arterial blood pressure was measured with a TechLine Z-40 electronic blood pressure monitor (Taiwan, China) on both arms, and the arm showing the higher mean pressure level was used [25].

Fasting blood samples were collected and stored in freezer at -80°C for future analyses. HDL-c was determined by the accelerator selective detergent methodology (AHDL Flex Reagent Cartridge). LDL-c was analyzed directly using an automated enzymatic method for LDL precipitation (ALDL Flex Reagent Cartridge), and triglycerides were measured by an endpoint bichromatic colorimetric enzymatic method using the TGL Flex Reagent Cartridge. Plasma glucose was detected by an adaptation of the hexokinase/glucose-6-phosphate dehydrogenase method using the GLUC Flex Reagent Cartridge. The HDL-c, LDL-c and glucose were determined in fresh blood samples.

### Accelerometer

Sedentary behavior (SB) and PA were evaluated using GT3X and GT3X+ accelerometers (Actigraph LLC, Pensacola, FL, USA) and the data were analyzed with the Actilife software (Actigraph v.6.11.7). The participants were instructed to use the accelerometer for seven consecutive days. The accelerometer was attached on the right side of the hip with an elastic belt and the participants were asked to remove it only for sleeping, showering or performing activities involving water (e.g., water gymnastics, fishing, and swimming). Participants showing low mobility (wheelchair users, bedbound individuals, and those with walking difficulties) were considered ineligible. Monitoring and quality control of accelerometer use were performed by telephone contact on the second and fifth day of use. Consecutive values of zero (with a tolerance of two minutes) over 60 minutes or more were interpreted as a period of non-use and were excluded from the analysis. The data were only considered valid when the participant had used the accelerometer and had accumulated a minimum number of records over 4 days of use during the week (10 h/day) including one weekend day (8 h/day). The mean SB and PA values were calculated using the following cut-off points: SB (0–99 counts/min), LPA (100–1,951 counts/min), MVPA (≥ 1,952 counts/min) and vigorous physical activity (VPA) (> 5,725 counts/min) [25]. These cut-points are commonly used in studies with older adults [26,27], so we decided to use them to compare our results with those of other studies. Individuals were considered to achieve PA recommendations if they had participated in MVPA for 30 min or more per day [28]. Participants reporting ≥ 150 min/week of MVPA measured with an accelerometer were classified as active and those performing ≥ 10 to 149 min/week of MVPA as insufficiently active [28]. All data were analyzed as minutes/day to adjust for the number of days when the device was used.

### Covariates

The covariates were selected based on previous studies [11,29–32]. Sociodemographic data were collected during a face-to-face interview at the participant’s home. The following covariates were used: gender (female and male), age group (60 to 69, 70 to 79, and ≥ 80 years), and education level (without formal education, 1–4, 5–11, and ≥ 12 years of schooling). Smoking was identified by asking, “Do you smoke or did you smoke cigarettes?” The answer options were: no, smoked and stopped, and current smoker. Alcohol use was assessed by the Alcohol Use Disorders Identification Test (AUDIT) [33] and was classified according to the number of doses consumed: non-consumer, non-abusive consumer, and abusive consumer. The presence of self-reported comorbidities was identified by the question “Did a doctor or health professional say that you have…?” The answer options were spinal or back disease, arthritis or rheumatism, bronchitis or asthma, tuberculosis, cirrhosis, osteoporosis, chronic renal failure, cancer, diabetes, hypertension, heart or cardiovascular disease, and stroke. The results were categorized as none, one or two, and three or more comorbidities.

The simultaneous effect of SB and PA behavior was determined by considering excess SB (≥ 10 h/day) and active physical activity (≥ 150 min/week of MVPA). The participants were thus classified into four groups: a) active (≥ 150 min/week of MVPA) and less sedentary (< 10 h/day); b) active (≥ 150 min/week of MVPA) and more sedentary (≥ 10 h/day); c) less active (< 150 min/week of MVPA) and less sedentary (≥ 10 h/day); d) less active (<150 min/week of MVPA) and more sedentary (< 10 h/day). Although there are no established criteria in the literature to classify the SB through the time in hours that is spent in this activity, studies have shown that the average time spent by older people in SB varies from 8.5 to 9.6 hours [22,34]. Then, after analyzing the distribution of our data, we decided to consider the cutoff point of 10 hours per day, being supported by the findings previously described.

### Data analysis

Quantitative variables are described by measures of central tendency and dispersion (mean and standard deviation), and proportions and respective confidence intervals (95%CI) were calculated for the qualitative variables. The normality of the outcome variables was assessed by determining asymmetry, kurtosis and coefficient of variation and by the Shapiro-Wilk test. A multiple linear regression model was used to estimate the association between the outcomes (WC, SBP, DBP, HDL-c, triglycerides, plasma glucose) and exposure variables (SB, LPA, MVPA), reporting the regression coefficients (β) and their respective 95%CI. A level of significance of 0.05 was adopted. Possible confounders were included simultaneously in the multivariate analysis of each outcome and exposure, regardless of the p value. All analyses were performed using the sample weights, which consider the effect of sample design by conglomerates. The Stata/SE 12.0 for Windows software (Stata Corp., College Station, USA) was used for analysis.

## Results

The sample included 425 older adults (59.8% women) with valid accelerometer and cardiometabolic data, who ranged in age from 63 to 92 years, with a mean age of 73.9 (95%CI: 73.5–74.4). Overall, the participants had 1 to 4 years of schooling (31.4%) and there was a predominance of non-smokers (59.2%), non-consumers of alcohol (49.6%) and subjects with three or more comorbidities (56.1%) (Table 1).

**Table 1:** Characteristics of participants with valid accelerometer data. EpiFloripa Idoso Study, Florianópolis, Brazil, 2015, (n = 425).

Variables	n	%
Gender		
Male	160	40.2
Female	265	59.8
Age group		
63–69	191	45.4
70–79	178	39.4
≥ 80	56	15.2
Years of Schooling		
Without formal education	22	4.8
1–4 years	149	31.4
5–8 years	71	17.5
9–11 years	65	18.4
12 or more years	118	27.9
Smoking		
No	267	59.2
Smoked and stopped	131	33.5
Current smoker	27	7.3
Alcohol use		
Not consumer	227	49.6
Non-abusive consumer	124	29.5
Abusive consumer	74	20.9
Comorbidity^£^		
0	27	7.0
1–2	153	36.9
3 or more	245	56.1
***Cardiovascular and metabolic variables***		**Means±SD**
Waist circumference (cm)	425	95.1±12.8
Systolic blood pressure (mmHg)	423	149.6±22.5
Diastolic blood pressure (mmHg)	423	84.8±12.7
HDL-c (mg/dL)	421	50.3± 13.2
Triglycerides (mg/dL)	414	119.4±50.2
Fasting plasma glucose (mg/dL)	421	103.4 ±22.4
***Accelerometer-derived variables***		
Sedentary behavior (min/day)	425	630.1±106.1
Light physical activity (min/day)	425	310.7±100.2
Vigorous physical activity (min/day)	425	0.58±2.6
MVPA (min/day)	422	19.2±18.8
Total MVPA (min/week)	425	115.7±117.7

HDL-c = High-density lipoprotein cholesterol; MVPA = moderate to vigorous physical activity; SD = Standard deviation.

^£`^Comorbidities: spinal or back disease, arthritis or rheumatism, bronchitis or asthma, tuberculosis, cirrhosis, osteoporosis, chronic renal insufficiency, cancer, diabetes, hypertension, heart or cardiovascular disease, and stroke.

Table 2 shows the results of linear regression between PA, SB and cardiometabolic risk variables. Each minute spent in SB was significantly associated with a reduction of 0.03 mmHg (95%CI: -0.05; -0.01) in SBP and of 0.02 mg/dL (95%CI: -0.02; -0.01) in HDL-c after adjustment. Light physical activity was not associated with any cardiometabolic risk markers after adjustment for gender, age, schooling, smoking, alcohol use, comorbidities, total MVPA and sedentary behavior. Beneficial associations of MVPA with WC (β = -0.15; 95%CI: -0.24; -0.05), SBP (β = -0.18; 95%CI: -0.32; -0.04), HDL-c (β = 0.10; 95%CI: 0.01; 0.18) and plasma glucose (β = -0.18; 95%CI: -0.33; -0.02) were observed after adjustment, but not with DBP (β = -0.03; 95%CI: -0.11; 0.06) or triglycerides (β = -0.05; 95%CI: -0.40; 0.29). Similarly, VPA was associated with higher levels of HDL-c (β = -0.35; 95%CI: 0.14;0.54), but it was not related to the other cardiovascular risk markers.

**Table 2:** Multiple linear associations of sedentary time and physical activity with cardiometabolic variables in Brazilian older adults. EpiFloripa Idoso Study. Florianópolis, 2015.

Variables	n	Sedentary behavior (min/day)		Light physical activity (min/day)		MVPA (min/day)		VPA (min/day)	
		β (95% CI) ^a^	*p*	β (95% IC) ^b^	*p*	β (95% IC) ^c^	*p*	β (95% IC) ^c^	*p*
WC (cm)	422	0.02 (-0.01;0.03)	0.079	0.13 (-0.08;0.34)	0.237	**-0.15 (-0.24; -0.05)**	**0.002**	0.02 (-0.39;0.42)	0.940
SBP (mmHg)	420	**-0.03 (-0.05;-0.01)**	**0.017**	0.02 (-0.36;0.40)	0.933	**-0.18 (-0.32;-0.04)**	**0.009**	-0.13 (-0.48;0.22)	0.455
DBP (mmHg)	420	-0.01 (-0.01;0.01)	0.672	0.05 (-0.19;0.30)	0.663	-0.03 (-0.11;0.06)	0.542	0.35 (-0.16;0.86)	0.180
HDL-c (mg/dL)	418	**-0.02 (-0.02;-0.01)**	**0.019**	-0.08 (-0.32;0.17)	0.538	**0.10 (0.01;0.18)**	**0.027**	**0.35 (0.14;0.54)**	**0.001**
Triglycerides (mg/dL)	411	0.04 (-0.01;0.08)	0.179	-0.75 (-1.80;0.28)	0.152	-0.05 (-0.40;0.29)	0.760	0.13 (-2.40;2.66)	0.920
Fasting plasma glucose (mg/dL)	418	0.01 (-0.01;0.03)	0.229	-0.02 (-0.29;0.2)	0.907	**-0.18 (-0.33;-0.02)**	**0.020**	-0.28 (-0.65;0.11)	0.158

WC = Waist Circumference; SBP = Systolic Blood Pressure; DBP = Diastolic Blood Pressure; HDL-c = High density lipoprotein cholesterol; MVPA = Moderate to Vigorous Physical Activity; VPA = Vigorous Physical Activity.

^a^ Adjusted by gender, age group, schooling, comorbidities and total MVPA.

^b^ Adjusted by gender, age group, schooling, smoking, alcohol use, comorbidities, total MVPA and sedentary behavior.

^c^ Adjusted by gender, age group, schooling, smoking, alcohol use, comorbidities and sedentary behavior.

**Fig 1:**
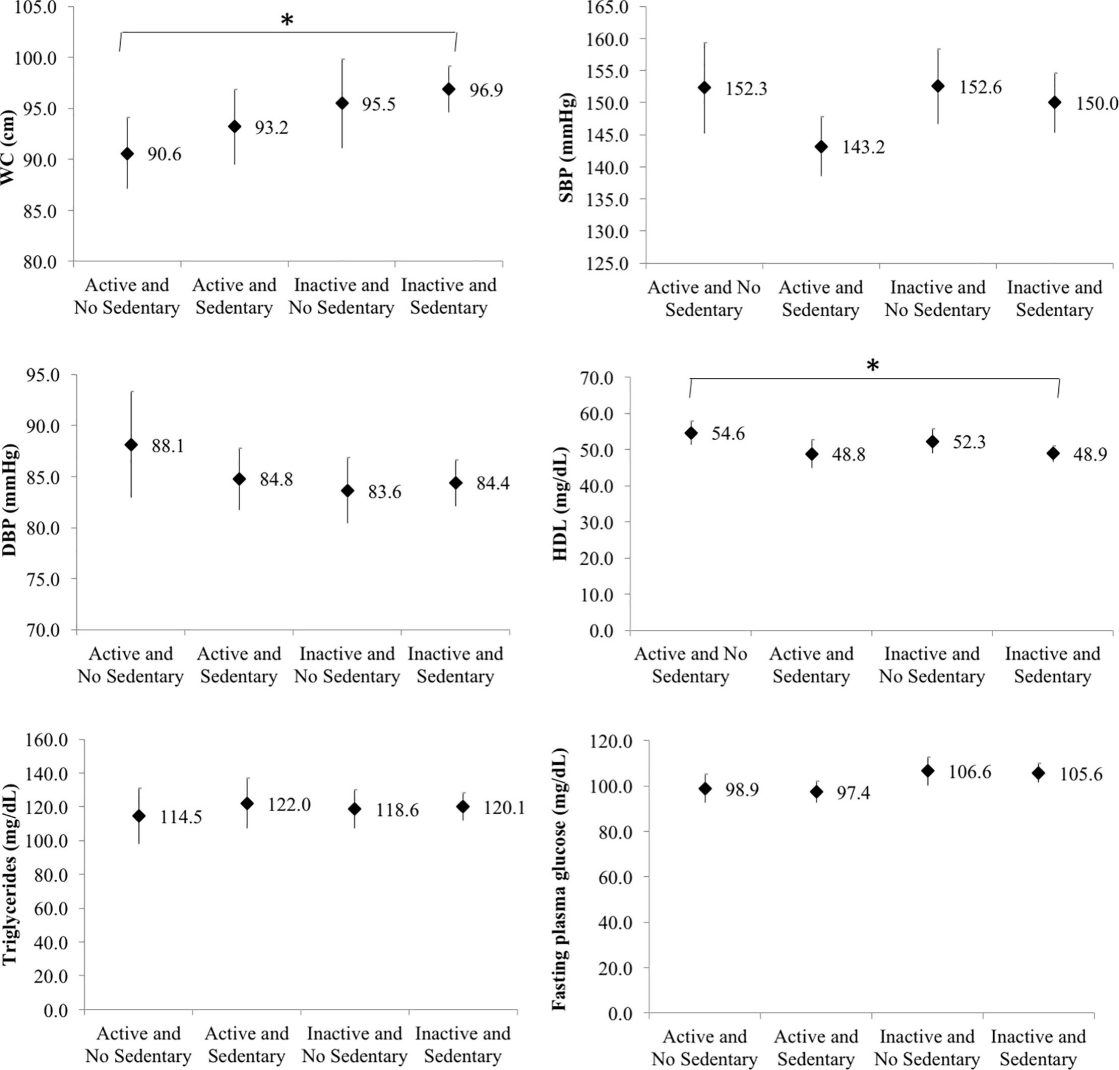
Means values of cardiometabolic variables according to the simultaneity of physical activity and sedentary behavior in Brazilian older adults. **Florianópolis, Brazil, 2015.** WC = Waist Circumference; SBP = Systolic Blood Pressure; DBP = Diastolic Blood Pressure; HDL = High density lipoprotein cholesterol. * p < 0.05.

The Fig 1 shows the mean cardiometabolic risk markers according to the simultaneous presence of recommended PA (≥ 150 min/week) and excess SB (≥ 8 h/day). Physically inactive and sedentary individuals had a greater mean WC (96.9 cm) and lower HDL-c (48.9 mg/dL) than physically active and non-sedentary subjects (90.6 cm and 54.6 mg/dL). No differences were observed in the other cardiometabolic risk markers.

## Discussion

The results show a significant association between different intensities of PA and SB and markers of cardiometabolic risk in older adults. A longer time spent in SB was found to be associated with lower HDL-c and SBP levels, regardless of the practice of PA. On the other hand, a longer time spent in MVPA was associated with lower WC and blood glucose levels and higher HDL-c concentrations regardless of sedentary behavior, while a longer time spent in LPA was not associated with any cardiometabolic risk markers. In addition, the simultaneous presence of excess SB (> 10 h/day) and failure to achieve PA recommendations (< 150 min/week of MVPA) was related to greater WC and lower HDL-c levels. These results reinforce available evidence that the effects of SB and PA are independent [35], acting differently on cardiometabolic risk markers in older adults according to the time spent in SB and PA of different intensities. Thus, in addition to the characteristics of this population, it is important to clarify the role of synergistic effects of excessive sedentary time and low levels of MVPA on outcomes such as abdominal obesity and HDL-c.

We observed that a longer time spent in SB was associated with lower HDL-c and SBP levels. The relationship between SB and lower HDL-c is consistent with the literature [31,36–38]. A study investigating adults and older adults found that an increase of 100 minutes in SB was related to a reduction of 0.13 mmol (95%CI: -0.18; -0.080) in HDL-c [37]. Similarly, another study conducted on 12,083 subjects between 18 and 74 years of age detected a significant linear trend between the quartiles of SB and mean HDL-c levels [31]. Evidence indicates that lipoprotein lipase acts as a key enzyme in the regulation of lipid metabolism which, in turn, is affected by low levels of PA and SB, and may be related to changes in HDL-c levels [39]. Thus, reducing the time in sedentary activities in the elderly population may be an important strategy to increase HDL-c levels. Decreasing the time spent in SB, older adults would be more exposed to PA. This promote an effective action on lipoprotein lipase and may increase HDL-c [40,41]. An unexpected finding was the association between longer time spent in SB and lower SBP. A negative, although not significant, association between SB and SBP has also been reported in some studies [30,32,42]. On the other hand, while some studies demonstrated a significant positive association between SB and SBP even after adjustment for MVPA [37,38,43], others were unable to identify such association [29,31,32,36,44–46]. Thus, the mechanisms underlying this relationship remain unclear.

Regarding LPA, greater participation in these activities was not associated with any cardiometabolic risk factor after adjustment for gender, age group, schooling, smoking, alcohol use, comorbidities, total MVPA and sedentary behavior. Indeed, the association between LPA and cardiometabolic risk markers is not consistent. In some studies on older adults, LPA was associated with lower SBP [43] and higher HDL-c levels [47], while another study found no such association with SBP [35]. Some of these inconsistencies may be related to the thresholds used to define LPA, which range from static activities such as standing and stretching to more dynamic activities such as gardening and walking [47]. Thus, it appears that the health benefits attributable to LPA depend on the type of intensities that compose it [47]. On the other hand, studies that have found associations with cardiometabolic risk markers and LPA have not adjusted the analyzes for sedentary behavior [35,43,47]. This could be one of the reasons for the different results found in our analyzes, which shows that this association between LPA and cardiometabolic risk markers is not independent of sedentary behavior. It seems likely that sedentary behavior has a significant and negative impact under cardiometabolic risk markers, which could be mitigated by MVPA and VPA, but not by LPA.

With respect to MVPA, the greater participation in this type of activity was associated with lower WC, lower blood glucose and SBP levels, and higher levels of HDL-c. These results corroborate the literature [48,49]. A meta-analysis showed that the increase in HDL-c and the reduction in blood glucose could be attributed to the practice of MVPA [8]. Other studies also demonstrated the role of MVPA in lower WC [50,51], blood glucose [50,52] and SBP [52,53] and in higher levels of HDL-c [51,54,55]. The benefits of MVPA for cardiometabolic risk markers are related to the increased energy expenditure in this activity [30], increased glucose uptake by GLUT4 in skeletal muscle, decreasing blood glucose [56,57], and reduced arterial stiffness and increased vascular relaxation, which contribute to blood pressure control due to the hypotensive effect of the exercise [53,58]. The MVPA increases the levels of enzymes responsible for the removal of triglycerides and free cholesterol from the bloodstream, as well as the increased production of HDL-c, which promotes reverse cholesterol transport. Thus, MVPA causes a decrease in the flow of free fatty acids to the liver associated with a decrease in VLDL production [10]. In this respect, although the prevalence of MVPA is low in older adults, this intensity provides greater protection for the cardiovascular and metabolic systems. Scientific evidence demonstrates that MVPA is more effective in achieving health benefits [50], as observed in this study in which the execution of this PA intensity provided more beneficial associations with the cardiometabolic risk markers. Thus, MVPA should be encouraged in older adults to increase the levels of this PA intensity and to ensure greater health benefits in this growing population group.

In turn, the time spent in VPA was much lower, which may have contributed to the absence of several associations which were identified with the MVPA. Thus, further analyses are needed to identify the specific contribution of VPA to cardiometabolic risk markers in the older adults.

The simultaneous presence of excess SB and insufficient levels of MVPA were associated with greater WC and lower HDL-c levels. Similar results have been reported in studies conducted in high-income countries such as Belgium, the United States, and the United Kingdom [11,36,44]. Among the studies involving specific samples of older adults [11,36,44], the proportion of time spent in SB was associated with metabolic syndrome, regardless of PA. This independence demonstrated in the literature may be due to the association between SB and unhealthy eating patterns (higher energy intake by sedentary individuals). This fact may explain the positive association of sedentary time with abdominal obesity [59] and lipid profile [60], regardless of the practice of MVPA. However, a recent study [38] showed that MVPA attenuated the associations between SB and cardiometabolic risk factors in a composite sample of adults and older adults. This finding can be explained by the fact that PA provides protection for the cardiometabolic system [61], but operates in ways other than SB [11,62]. Nevertheless, it should be noted that the benefits of PA do not compensate for the harmful effects of SB [35]. Considering that older adults spend large amounts of time in SB, programs aimed at reducing the incidence of CVD in this population should address both physical inactivity and SB.

Possible limitations of this study include its cross-sectional design, which does not allow us to establish causal inferences between SB and PA levels and cardiometabolic risk markers. The loss to follow-up should be considered in performing the clinical examinations since this caused the sample to be no longer representative of the initial population by consisting of older adults with better health conditions than those who refused to participate in this stage. This may imply underestimation of the results reported. Another limitation is the fact that possible confounding factors such as body mass index and the use of antihypertensive drugs, antidyslipidemic drugs and antidiabetics drugs were not included in the analyses. Furthermore, in multivariate analyzes for VPA it is important to consider that only 10% (n = 44) of the adults older performed this physical activity, of which only nine per cent (n = 4) performed more than 10 minutes on day. The loss of some associations may be related to the small number of people who have achieved this level of physical activity, which may decrease the study's statistical power.

In addition, we reinforce the need of additional studies that consider the cutoff point of 10 hours in sedentary behavior or investigate different cutoff points to evaluate the simultaneity of this behavior with the practice of MVPA.

However, a strength of the study is the use of objective measures to assess SB and PA, which are more accurate than self-reported data [22]. Furthermore, a series of measurements were performed in the laboratory following procedures validated in the international literature, which permits comparison with other studies. Standardized questionnaires were used for data collection and periodic quality analysis of the information obtained was performed.

In conclusion, this study demonstrated the association between PA intensities, SB and cardiometabolic risk markers in a population at high risk for CVD. Despite the benefits provided by the different intensities of PA, the simultaneous presence of SB and low PA level was associated with poor cardiometabolic risk markers. The pattern of SB was found to be determinant for the cardiometabolic parameters of older adults. Although programs encouraging the participation in PA are available, it is necessary to develop measures designed to reduce the prevalence of SB in this population. Further studies are needed to clarify the relationship between LPA and cardiometabolic risk markers, considering also the sedentary behavior, and to establish effective interventions to reduce SB in the older adult population.

## Supporting information

S1 Appendix Markers of cardiometabolic risk, physical activity, sedentary behavior, and sociodemographic variables and life habits of Brazilian older adults. Florianópolis, Brazil, 2015.
(XLSX)
